# Knowing, Being and Becoming a Person-Centred Nurse Leader: Findings from a Transformative Professional Development Programme

**DOI:** 10.3390/nursrep14040230

**Published:** 2024-10-23

**Authors:** Clare Cable, Tanya McCance, Brendan McCormack

**Affiliations:** 1 Queen’s Nursing Institute Scotland, Edinburgh EH1 2EL, UK; clare.cable@qnis.org.uk; 2 Institute of Nursing and Health Research, Ulster University, Belfast BT15 1AP, Northern Ireland, UK; tv.mccance@ulster.ac.uk; 3 Sydney Nursing School, Faculty of Medicine and Health, University of Sydney, Camperdown, NSW 2006, Australia

**Keywords:** community nursing, leadership, transformative practices, person-centred practice, collaborative inquiry

## Abstract

*Background/Objectives* Leadership is central to the development of effective workplace cultures and as such should be viewed as a practice that is relational, exercised through a process of mutual and reciprocal influence. Person-centred leadership is an approach to leadership that supports a way of being that is authentic, prioritising values lived out in action. However, there is an increasing recognition that leadership development has not been impactful in relation to workplace culture. This paper reports on the ongoing evaluation of an innovative development programme (Queen’s Nurse Development Programme), the overall aim of which was to illuminate the participants’ experiences of engaging in transformative learning and development and identify the technical and transformative outcomes arising. The programme focused on developing leadership capacity for societal change and maximising the health impact of community nursing leaders. *Methods* The methodological framework for evaluation was underpinned by a Collaborative Critical Creative Inquiry. Twenty community nurses were selected to undertake the programme during 2020. The collection and analysis of data was consistent with the Collaborative Critical Creative Inquiry and was conducted as a one-day workshop, with participants engaged in a cycle of creative hermeneutic analysis. *Results* A total of seven themes were identified, including: sense of belonging; personal growth; developing new skills; finding voice; importance of self-care; and creating a safe place. This illuminated how the transformative learning and development processes within this programme were experienced and how these enabled participants to explore how they influence their practice and workplace. It is the journey with self that generates a sense of belonging and enables personal growth and the ability to care for self and others. *Conclusions* The key learning from this innovative development programme is the importance of focusing on the attributes of practitioners and the key building blocks for knowing, being, and becoming a person-centred practitioner.

## 1. Introduction

Person-centredness is a global movement in healthcare because it reflects the importance of keeping people at the centre of healthcare systems. It prioritises the human experience and places compassion, dignity and humanistic caring principles at the centre of planning and decision making and is translated through relationships that are built on effective interpersonal processes. A unique perspective of person-centredness can be viewed through the lens of the person-centred practice framework developed by McCance and McCormack [1], which is a theoretical model developed from practice, for use in practice. This framework enables the articulation of the dynamic nature of person-centredness, recognising complexity at different levels within healthcare systems, and identifies healthfulness as THE outcome arising from the development of person-centred workplace cultures. Healthfulness means ensuring that the environment in which healthcare is experienced places individual health and the wellbeing of all persons as the core concern.

Leadership is central to the development of effective workplace cultures, and as such, should be viewed as a practice that is relational, exercised through a process of mutual and reciprocal influence [2]. Person-centred leadership is described as a “complex, dynamic, relational and contextually embedded practice that fosters healthful relationships and growth” [3]. This is an approach to leadership that supports a way of being that is authentic, prioritising values lived out in action. Enghiad et al. [4], in a recent integrative literature review exploring clinical leadership in long term care, confirmed the influence of a leader’s beliefs and values on the motivation for providing high-quality care. Therefore, it is not surprising that leadership development is complex and context dependent, particularly so for nurses who are directly involved in providing care. In a recent systematic review focusing on nurses’ clinical leadership in a hospital setting, Guibert-Lacasa and Vazquez-Calatayud [5] concluded that interventions for leadership skill development required cognitive, interpersonal and intrinsic competences. They proposed the development of multicomponent programmes to address these aspects, including psychological empowerment skills, emotional intelligence and critical reflexivity.

There is an increasing recognition that leadership development has not been impactful in relation to workplace cultures. Some of the reasons for this were presented in a discussion paper by Edmonstone [6] and included assumptions that leadership is context free and there is over-emphasis on competence rather than capacity. Furthermore, Edmonstone suggests there is a focus on the development of individual leaders as opposed to leadership and highlights the importance of leadership development for social capital and the need to view leadership as a practice-based activity. The programme described in this paper was established with this challenge in mind and deliberately set out with a focus on developing leadership capacity for societal change and maximising the health impact of community nursing leaders. So, the emphasis shifted from leadership development to a transformative development programme with a focus on individual transformation for social change in communities with a focus on health and wellbeing.

The Queen’s Nursing Institute Scotland (QNIS) is a charitable organisation that is founded on a commitment to enable nurses to make a positive difference in communities, with a focus on maximising the potential of nursing for social justice. In recognition of the need to develop enhanced ‘change-making’ skills in community nurses, the organisation designed a programme underpinned by the QNIS Excellence Profile, which focuses on helping participants to develop excellence qualities relating to: inspiring others by making a difference; inspiring others with tenacity and resilience; inspiring others by bringing people with them; and inspiring others with humility and reflection. Two complementary theoretical frameworks were chosen to inform the programme design: theory U [7] and the person-centred practice framework [1]. In a previous paper, McCormack et al. [8], described the programme in more detail and the links between theory U and the person-centred practice framework:

“Like Theory U, person-centred practice requires us to connect with our inner selves as human beings with feelings, emotions, thoughts and desires that guide us as persons. It is the sum of these that guide us towards ‘that which really matters’ and a connection with our unique humanness as persons—our embodied knowing” (p. 4).

To co-create healthful cultures, the Queen’s Nurse Development Programme invites participants on a journey of awareness-based systems change, which is transformational. This is beautifully captured in theory U, which sets out the journey that we are to make, individually and collectively, to sense the emerging future. It requires learning new ways of sensing, using all five senses, and a deepening awareness. Theory U sets out a set of principles that can be presented as five movements that follow the path of the U, including: co-initiating, co-sensing, co-presencing, co-creating, and co-evolving. The programme is structured as a nine-month journey of discovery, weaving together residential workshops, individual co-active coaching (through an individually assigned coach) [9], with an issue for development, which the participants explore as they become their best selves as skilled facilitators of change.

This programme began in March 2020, the year when COVID-19 redefined ways of working. It has been recognised that during the pandemic, community nurses went above and beyond, continuing to care for the most vulnerable and frail citizens wherever they lived. Some nurses were working in full PPE for over a year, sweating through long shifts encased in plastic, donning and doffing dozens of times a day, struggling with flimsy aprons outside in the wind and rain, working behind visors and steamed up spectacles. Others spent hours on the phone on videoconferencing systems trying to connect with people who were isolated and anxious and on MS Teams negotiating to ensure the needs of their teams and communities were met. This made relationships more complex and brought significant stresses and challenges that impacted on leadership practices.

This paper reports on the ongoing evaluation of this innovative development programme and builds on the outcomes reported in an initial evaluation [8]. This paper provides insights into the impact of the programme, on individuals, their practice, and more widely within their teams and the communities where they work.

## 2. Methods

The overall aim for the programme evaluation was stated as follows:

To illuminate the participants’ experiences of engaging in transformative learning and development, and to identify the technical and transformative outcomes arising.

The methodological framework for evaluation was underpinned by a collaborative critical creative inquiry (CCCI) methodology. A CCCI methodology combines collaborative inquiry [10] with critical creativity [11]. Critical creativity combines being critical with being creative, i.e., integrating cognitive critique with creative practices. This framework is based on key theoretical and methodological assumptions that have a primary focus on human flourishing as both a process and outcome. The aim that guided this component of the evaluation was as follows:

To understand if and how the transformative learning and development processes experienced have enabled participants to create the conditions required for their continued flourishing as community nurses.

The focus was primarily on the impact of the programme on individuals, their practice, and their wider communities. More specifically, it focused on the following evaluation objectives as outlined in the evaluation framework:

1. Understand the influence of the programme in shaping and informing individual and collective development.
2. Determine the technical and transformative outcomes arising from participation in the programme.
3. Build an ongoing body of knowledge about transformative learning and development, its impact on individual and collective action, and the contexts that sustain it.

### 2.1. Selection of Participants

Twenty community nurses were selected to undertake this transformational development programme during 2020. Following nomination by the executive lead in their organisation, which ensures employer support from the outset, each nominee submitted a written application. A selection panel shortlisted the candidates to be invited for the second stage. Shortlisted candidates attended a selection day that included focus groups and multiple mini-interviews. The focus was on in-depth exploration of the quality of the match between each candidate and the Excellence Profile developed by QNIS.

### 2.2. Data Collection and Analysis

The data collected in this project consisted of three types of data, constituting the everyday inquiry records maintained by participants—creative expressions, reflective diaries and journals, and project notes of specific discussions. These methods are described in detail elsewhere [8]. Throughout the programme, the participants retained their own data in an ‘evidence folio’ provided by the programme leaders and were asked to bring their folio of evidence to the data analysis workshop. They were also asked to anonymise any data they wished to and redact any data they did not feel comfortable sharing with others. In this way, participants retained control of their data and its analysis. The analysis of data was consistent with CCCI and was conducted as a one-day workshop. The participants engaged in a cycle of creative hermeneutic analysis focusing on the key questions below:

(i) What impact has the programme had on your practice and workplace?
(ii) How did the programme content enable you to deal with uncertainty, anxiety and trauma through the pandemic?

**Figure 1:**
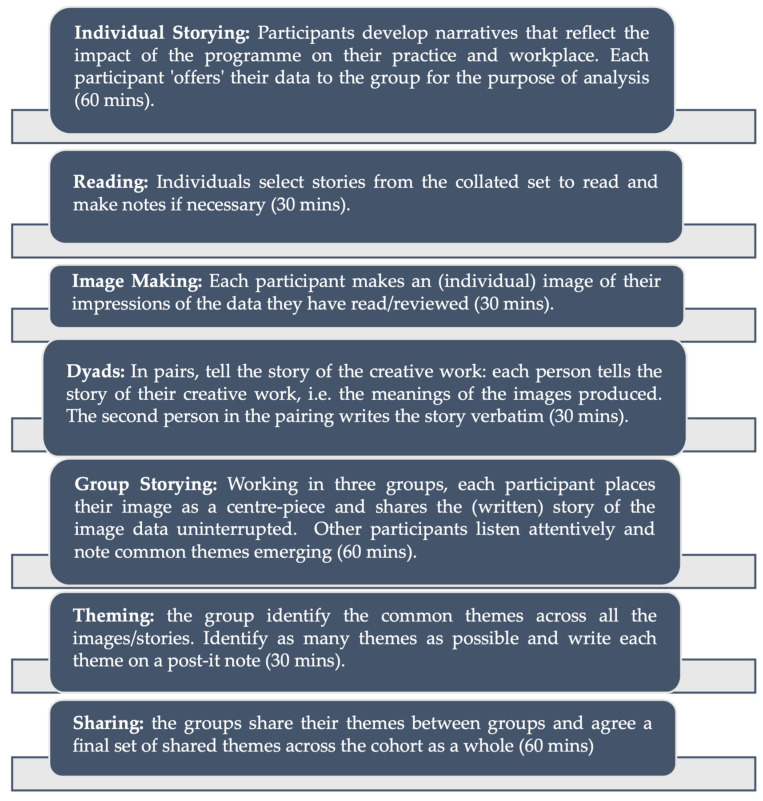
Overview of Collaborative Critical Creative Inquiry (CCCI) Methodology.

The critical creative hermeneutic process followed the steps outlined in Figure 1, which was adapted from the data analysis described in the Evaluation Framework [8]. Participants worked as a whole group (all 20 participants), in small groups of 6–7 people, in dyads and as individuals as they moved through the steps of data analysis. All of the data sources were revisited to provide a robust audit trail for the common themes identified through the critical creative hermeneutic process. A detailed data extraction process was developed and following review of the outcomes from this process, further refinement was undertaken by the authors of this paper and a final set of themes was generated as described in the results section of this paper.

### 2.3. Ethical Considerations

This collaborative enquiry was underpinned by a robust ethical framework that reflected the principles of person-centred research and was embedded throughout the delivery and evaluation of the programme, which, consistent with the underpinning values of collaborative inquiry and the shared values of the development programme, participants hold one another accountable for working with and adhering to these shared values. The key ethical considerations in undertaking this component of the evaluation focused on: ensuring voluntary participation; assuring anonymity and confidentiality for participants where appropriate; and ensuring the psychological safety of participants throughout the process. Participants were informed of the purpose of the evaluation workshop in advance, enabling individuals to decide if they wished to participate. Ways of working had already been established for the group and were also used as the basis for conducting the workshop. This addressed issues of negotiating anonymity and confidentiality and ensuring participants engaged in self-care if the workshop revealed issues that were potentially distressing or upsetting. For these reasons, formal ethics approval was not sought for this evaluation, as was the case for the initial evaluation [8], the rationale being that evaluation is integral to the programme.

## 3. Results

**Figure 2:**
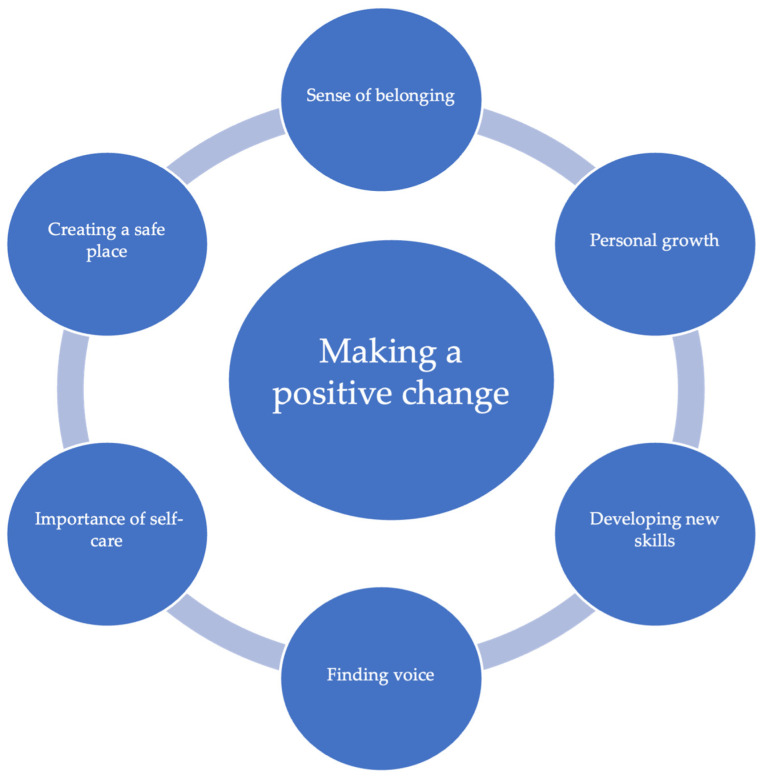
Overview of themes.

A total of seven themes were identified, which illuminated how the transformative learning and development processes within the Queen’s Nurse Programme were experienced and how these enabled participants to explore how they influenced their practice and workplace. The findings also provide insights into how the participants’ journeys were experienced in the context of the ongoing pandemic. The themes are presented in Figure 2, and each is discussed in turn below. The audit trail supporting the themes is drawn from the different stages of the CCCI methodology presented in Figure 1.

### 3.1. Sense of Belonging

The Queen’s Nurse Development Programme offered a space for participants to link with like-minded practitioners who understood the role of a community nurse. Being seen by others was central to this theme: “Others see us as different, we see each other” (Participant 4). Linked to this was the freedom to be their authentic self back in the workplace.

“It also allowed us as Queens Nurses not to wear a different mask. With all these tools allows a sense of togetherness and (other) staff a sense of involvement” (Participant 7)

For some, they felt isolated and had lost their identity as a nurse, but the programme provided an opportunity to reaffirm their passion for nursing and develop an appreciation of the breadth of skills and expertise held by community nurses, and the types of environments in which they work.

“But realising being part of a team of QNIS nurses, who always have your back, helped me become the leader I am today and the passion to continue to be the best I can be and to take others with me along the way”. (Story 1)

### 3.2. Personal Growth

The opportunity for participants to explore self in the context of their own professional development was central to the experience within the Queen’s Nurse Development Programme. The gift of being able to take time out, to slow down and to reflect enabled exploration of what was important and how this related to self-knowing. Participants talked about the different roles they undertook, personally and professionally, but self knowledge offered clarity on what was important and the ability to be authentic.

“The program starts a deep reflection within who am I how can I make a difference. Speakers who inspire, strong people who know who they are and are “compassionate” A question to develop and probe into my core. What is important to me, how can I influence, support, develop, be true to myself, but open to opportunity as they arise”. (Story 9)

“Prior to becoming a Queens nurse, I had real struggles with my own identity and professional direction. Honestly not really knowing who “the real me” was, and feeling like I wore different “masks” for my different roles as: mum, wife, nurse, friend, colleague, daughter … I am now a confident practitioner who has greater understanding of the impact I have in others. Importantly, I no longer feel like I need to wear masks, I have an understanding of who I am and realise I now quite like who that person is”. (Story 5)

Having a personal coach to facilitate this process was a further enabler in the process, and supported exploration of self, in the context of being a community nurse.

“Time out for me to stand back and reflect. I have been 10 years in this post and hit the ground running and now it’s like my treadmill emergency button has been pulled with stillness and calmness. Leisurely walks picking flowers. This was not easy to begin with trying to restart the treadmill at times. Wow! Permission to focus on me! Coaching? My own coach!” (Story 4)

### 3.3. Developing New Skills

The development of new skills was frequently referenced by participants. Elements core to the programme included reflection and deep listening. The development of these skills was directly connected to the ability to approach issues differently in the workplace. One participant described how she worked differently to support her team during the pandemic, drawing on the skill of deep listening. Creating a safe space to engage in meaningful dialogue, focusing on issues that were important to the team, led to a more bonded team dynamic.

*“I very quickly recognised, what I had to do, it was my responsibility as deputy manager at the time to use what skill I had learned the week before with QNIS to support my staff team. The first thing I done was set up twice weekly huddle meetings. This allowed me a platform to create a safe space for staff to attend. The staff initially thought this was a space to receive updates about the pandemic and while it did do that, I also had another agenda—to use one of the practises I had learned at the beginning of my QNIS journey. I began by truly and deeply listening to the staff each week learning what they were feeling and using the time to enable them to fully express themselves where they had my full attention. This enabled me to support them to find their own coping strategies. We spent time talking at length. I showed my own fears and worries in a professional manner, this helped us all bond as a team”. (Story 13)*

Using skills from the programme, however, was not always easy for participants. One of them describes a sense of distrust from colleagues when she tried a different approach, which was fuelled by lack of motivation and low morale. The importance of using processes that enabled collaboration with all members of the team was an enabler in achieving a positive outcome.

“On reflection, there are a lot of things I would have done differently, quicker, in a more meaningful way … Using the skills, I had learned at the QNIS programme was difficult at first. Because of staff’s experiences, they were fearful, did not want to put trust in me and lacked motivation and enthusiasm for their work. It took a year to build the team back up and “bring them with me”. With support from our new manager, I used deep listening, reflection, collaboration and sharing my vulnerabilities with them to allow them to start to trust again and open up”. (Story 16)

### 3.4. Finding Voice

Many participants felt that they had found their voice as a result of engaging with the Queen’s Nurse Development Programme. This was particularly important in the context of the pandemic and its impact on staff morale and the need to create a supportive working environment.

This was linked to having the confidence to speak up and to be heard, and to challenge when decisions were being made that did not prioritise people.

“But it also gave me my voice to go back and challenge decisions that were being made. At this time staff were being deployed into areas where they were not required, our service was struggling as a result”. (Story 6)

Those who found their voice described the positive impact on the workplace.

“I have found my voice and no longer struggle to tell the people how I am feeling. I prioritise myself in order to get the best out of myself and my staff and this directly affects patient care, staff well-being and a safe workplace culture”. (Story 7)

Linked to the theme of finding voice was the sense of being brave and taking risks. Examples of being brave provided by participants often related to delivery of the service and in response to the needs of patients, families and of staff. In these situations, the context was often challenging, exacerbated by the pandemic, requiring courage to voice concerns and take positive action.

“I carried on with my journaling and coaching and self-care practices. Through my reflection it hit me how fortunate I had been to have just been immersed in QNIS in Balbirnie pre-lockdown. I had been trying to raise an issue with regards waiting times and a review of service for the last 4 years. I now had time to prepare and present this again. Lots of questions. Is this wrong at a time when people are immersed in managing COVID. So, I did it, resubmitted it again! To my amazement it was approved and at last those that need to have this awareness were activated in reviewing the service and making it fit for purpose and robust”. (Story 4)

### 3.5. Importance of Self-Care

Fundamental to the Queen’s Nurse Development Programme is the necessity to care for self. The development of this awareness and tools to support the ability to engage in self-care were highly valued by participants. It underlined the importance of being able to look after self in order to show care towards others.

“Commencing the QN programme in March 2020, I felt guilty at being away from the workplace. I didn’t feel worthy of my nomination. Participating in all aspects of the programme has allowed me to recognise the importance of self care in order to achieve effective outcomes. And it wasn’t until December 2020 when I really understood and experienced the consequences of not looking after myself … My coach supported me to recognise that I am an emotionally connected leader that is able to tune into others. I have my values at the fore throughout my work/practice and I no longer let work/workload/stress define me”. (Story 7)

The importance of self-care became even more prominent in the midst of the pandemic. Participants offered examples of not only engaging in their own self care practices, but also supporting others to do the same. One participant describes how they implemented several practices with staff that were drawn for the programme and the positive impact experienced as a result.

“We looked at ways to focus on our own self care—putting our oxygen mask on so that we could then be there for our residents, bringing the best versions of ourselves every time we stepped into support with any resident care. We explored all kinds of ways as a team to practice self care. I even got them to do the leadership dance—some of the staff really enjoyed this while others thought I was a bit bonkers. Anyway, through doing this every week staff began to fell truly valued and appreciated, it enabled everyone to be heard and processes to be implemented to help everyone. I even implemented a wobble room for staff to go to if they needed this. The feedback from this was that staff felt this was a very safe space and were comfortable to use it because of the bonding we had through our weekly huddle meetings”. (Story 13)

### 3.6. Creating a Safe Place

Participants recognised that creating a safe space was an enabler to how they could engage with staff using the new skills they had developed in the Queen’s Nurse Development Programme. There was an appreciation of how the facilitators of the programme had created a safe space for them and how instrumental this was to their personal growth. This, in turn, equipped them to create a safe space for their team. One participant described how, in the midst of the pandemic, they were able to use the practice experiences in the programme to support their staff.

“The work environment had changed from a calm safe environment to what seemed like utter chaos with no rational decisions being made. Before the Queens Nurse programme I may have joined in on the panic but I had learned to breathe. I knew I had to create a space with my teams that we could all think logically about what support we could offer the acute sector whilst managing the public health nursing service in a way that could continue to support the children, young people and families and provide a safety net for our most vulnerable communities. I created a time to pause, gather my thoughts and use my voice to protect the health visiting service … The Queens Nurse programme not only taught me to stop and pause in the space but it gave me the tools to create that space in the workplace, in meetings to give time to people to ground themselves so that collectively we could all think clearly about the services, about the children and families and what we needed to prioritise”. (Story 6)

A sense of safety and the impact of a calm environment were identified as key elements of creating a safe space.

“Strength to ensure safety is paramount for my patients, advocating. But, also the new found confidence to lead in a different way supporting, nurturing staff to be the best they can be, be a visible leader, supporting, encouraging those around you, calming the situations”. (Story 9)

### 3.7. Making a Positive Change

Being able to make a positive change is evidenced throughout the previous six themes. Positive outcomes identified by participants focused on the enhanced wellbeing of staff, improved teamworking across both nursing and multiprofessional teams, and improvements in practice and service provision. The data supporting the other themes also supports this. The following offers feedback from participants on the specific outcomes they were able to achieve.

“As time went on staff and anxieties became less concerning, people started to learn this new way of life in care homes. We all worked together in a way that had never been done before and through time our weekly huddles came to a natural end. I am certain we would not have achieved this positive outcome if it had not been for the experiences and strategies, I had been taught by QNIS, these truly helped me lead my team during one of the most difficult times in my life”. (Story 13)

By giving me the combination of self-belief, bravery and the skills, I have been able to effect change—increasing community nursing establishment. More community nurse undoubtably is the biggest impact on my workplace. But also, the value those nurses feel. Someone cares—so we are safe to care. Someone listens so we are safe to listen. Someone held that space for us safely. So we can hold that space for someone”. (Story 11)

“The most valuable approach to achieving success was my determination to create a more collaborative multi-disciplinary team approach, by calling on the huge wealth of expertise of other community nurses and allied health professionals. Initial contact with Queen’s nurse colleagues led to immediate and enthusiastic offers of help and guidance—signposting me to learning resources and key services. As a result, I feel I now have a team of fantastic specialist nurses who I can work with to meet each patient’s needs most effectively, as well as gradually improving mutual trust and positive relationships with the care home staff”. (Story 12)

## 4. Discussion

**Figure 3:**
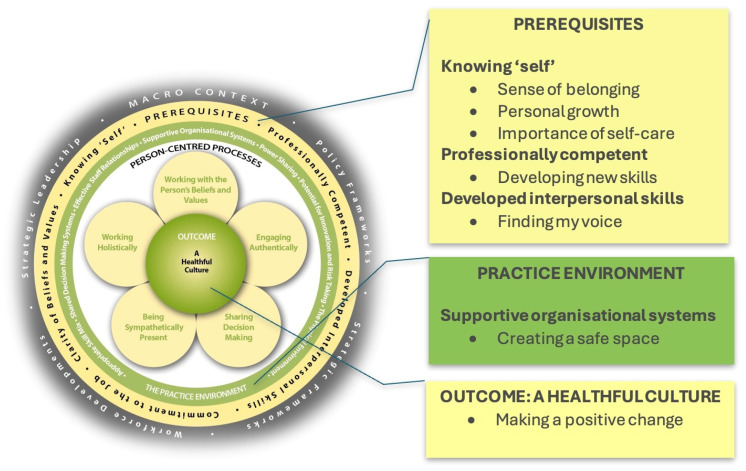
Themes mapped to the person-centred practice framework (McCance & McCormack 2021).

With the person-centred practice framework [1] being the theoretical underpinning for the Queen’s Nurse Development Programme, the findings will be placed in the context of the contemporary literature on person-centredness. Figure 3 illustrates how the themes map to the components of the person-centred practice framework. This shows how the transformational processes, brought about by engagement in the Queen’s Nurses Development Programme, has the potential to enable nurses to contribute to the development of person-centred practice. Five of the seven themes are positioned within the attributes domain of the framework, with one theme connected to the practice environment and the final theme connected to the creation of healthful workplace cultures.

The biggest impact from the Queen’s Nurse Development Programme aligns with the attributes domain and in particular ‘knowing self’. These findings reflect a way of being in the world, and as emphasised by McCance and McCormack [1], person-centredness requires attention to be paid to our being as persons. They define knowing self as “the way a person makes sense of his/her knowing, being and becoming through reflection, self-awareness and engagement with others” (p. 28). The themes identified under knowing self reinforce the importance of the four modes of being identified by McCance and McCormack [1]—being in relation, being in a social context, being in place and being with self. Being in relation emphasises the importance of relationships and the interpersonal processes that support the development of positive relationships and it is this that brings about a sense of belonging. Furthermore, personal growth reflects the fundamental need of being with self and gaining clarity about ‘that which really matters’ and being able to reveal this to others. Interestingly, Nolan et al. [12], in the senses framework that was developed in the context of relationship centred care, identified a sense of belonging as one element that was important in enhancing care environments. This included: enhancing relationships in the team through valuing and showing an interest in one another; being able to confide in people you trust; and feeling part of a valued group who share similar values and beliefs that connect you. This aligns to the sense of belonging described by the participants in this study.

Central to the Queen’s Nurse Development Programme are practices that support self-care and personal well-being. Practices learnt in the programme included: being in the body; activating a mindful awareness; embracing stillness through meditation; and the discipline of journalling, through, for example, creating a gratitude list or wellbeing list or recounting the experience of going for a reflective walk (QNIS 2021). These practices added a diversity to the skill set of participants and their use enabled them to function differently in the practice environment. The importance of self-care has been explored in the literature [13,14] but has become more prominent as a consequence of the pandemic [15]. Furthermore, in a systematic literature review, Lee et al. [16] illustrated the nurses’ capacity for compassion and the development of skills focusing on self-compassion, self-reflection and self-awareness. The findings from this study are similarly reflected in a review of the literature undertaken by Raab [17] who concluded that using mindfulness interventions for health care workers can reduce perceived stress and increase the effectiveness of clinical care.

The opportunity for participants to develop competence and confidence in their interpersonal ability by finding their voice and speaking out when it was important for patient care is directly related to developed interpersonal skills. McCance and McCormack [1] describe this attribute as “the ability of the person to communicate at a variety of levels with others, using effective verbal and non-verbal interactions that show personal concern for their situation and a commitment to finding mutual solutions” (p. 28). This also links to knowing self, and the importance of knowing and accepting who we are and the personal motivations that drive us, which can help improve interpersonal effectiveness.

The ability to create a safe space reflects the evidence base on psychological safety within healthcare environments. A psychologically safe environment is one where people feel able to focus on the underlying issues without threat of loss of self-identity or integrity [18]. Brown and McCormack [19] conducted an action research study to explore holistic facilitation as an approach to enable healthcare teams to critically analyse practice and enhance patient care. They highlighted the need to create psychologically safe spaces in environments where insufficient support, weak leadership and oppressed behaviours are apparent. Psychological safety enables individuals to feel safe to engage in difficult conversations and consider changes to practice. Supportive organisational systems as described in the person-centred practice framework [1] provide the conditions to create psychologically safe spaces for staff that enable people to flourish. Such systems “support initiative, creativity, freedom and safety of persons” (p. 28). Furthermore, psychological safety connects workplace culture to the health, resilience and well-being of individuals and teams [20], highlighting the relationship with the findings on knowing self.

One of the key findings in this study was the ability of the participants to bring about positive changes in the workplace as a direct result of engaging in the Queen’s Nurse Development Programme. This is an impactful finding and is a marker of an effective professional development programme that enables nurses to influence their workplace cultures. The centrality of leadership practices in the development of person-centred workplace cultures is well represented in the literature [21,22]. The positive outcomes presented from the findings of this study, such as enhanced wellbeing of staff and improved teamworking, reflect the development of healthful cultures, the core outcome arising from the development of person-centred practices in teams and organisations as identified in the person-centred practice framework. McCance and McCormack [1] define a healthful culture as one in which “decision making is shared, relationships are collaborative, leadership is transformational and innovative practices are supported” (p. 29). Use of the term healthful reflects a broader notion of health that embraces all dimensions of our being, and once again reflects the themes that were identified in relation to knowing self.

## 5. Conclusions

The relationship between a systematic approach to transformative professional development and the development of person-centred practices is at the core of this paper. However, what is most striking is the way in which participating in this programme enabled participants to embody the attributes that are a prerequisite for being person-centred. It is the journey with self that generates a sense of belonging, enables personal growth and the ability to care for self and others. This was complemented by the acquisition of new skills that focused on practices such as mindfulness, stillness, deep listening and finding voice. These practices added a diversity to the skill set of participants and their use enabled them to function differently in the practice environment by creating psychologically safe spaces for themselves and their teams. This in turn enabled them to make positive changes in practice, contributing to the development of healthful cultures. The key learning from this innovative development programme is the importance of focusing on the attributes of practitioners and the key building blocks for knowing, being and becoming a person-centred practitioner. This research adds to a growing body of evidence that highlights the importance of healthful workplace cultures for effective person-centred practices and staff wellbeing. However, we are conscious that the work articulated in this paper is intense and requires a high level of commitment from participants and supporting organisations. A key question in moving forward is how we can replicate such programmes at scale and enable these transformative processes to be normalized in all leadership development programmes that have the intent of developing person-centred practitioners and cultures.

## Data Availability

The datasets presented in this article are not readily available because they constitute the everyday inquiry records maintained by participants – creative expressions, reflective diaries and journals, and project notes of specific discussions. Accessing these data would breach confidentiality and anonymity of participants.

## Public Involvement Statement

There was no public involvement in any aspect of this research.

## Guidelines and Standards Statement

This manuscript was drafted against the guidelines for reporting participatory action research [23].
